# MorphoNet: An Interpretable Hierarchical Deep Learning Framework for Multi-Class Skin Lesion Classification Using Dermoscopic Morphology

**DOI:** 10.3390/bioengineering13090989

**Published:** 2026-08-27

**Authors:** Bradley Yao, Angela Jin, Huijuan Liu, Qiliang Li

**Affiliations:** 1 Weston High School, 444 Wellesley St, Weston, MA 02493, USA; 27yaob@weston.org; 2 Phillips Academy, 7 Chapel Ave, Andover, MA 01810, USA; angelajin131@gmail.com; 3 School of Advanced Manufacturing and Robotics, Peking University, Beijing 100871, China

**Keywords:** MorphoNet, skin cancer classification, EfficientNet-B4, ABCD rule, attention mechanism, CORAL domain alignment, dermoscopy

## Abstract

Accurate and interpretable classification of dermoscopic skin lesions remains challenging because lesion subtypes frequently exhibit overlapping visual characteristics, public datasets are highly imbalanced, and images acquired from different sources may present substantial domain variation. This study proposes MorphoNet, an interpretable two-stage deep learning framework for hierarchical skin lesion classification. A total of 16,845 dermoscopic images from HAM10000, ISIC 2019, and DERM12345 were integrated into eight diagnostic categories. In Stage 1, an EfficientNet-B4-based classifier distinguishes benign from malignant lesions. In Stage 2, separate classifiers identify four malignant and four benign subtypes. Quantitative asymmetry, border, color, and diameter descriptors are incorporated through an attention-guided feature fusion module, while correlation alignment regularization is used to reduce discrepancies in feature distribution across the three data sources. Experiments were repeated using three random seeds. MorphoNet achieved accuracies of 94.01 ± 0.50%, 89.05 ± 0.51%, and 91.84 ± 0.41%, with corresponding F1-scores of 93.70 ± 0.55%, 84.00 ± 1.00%, and 87.97 ± 1.13% for benign–malignant screening, malignant subtype classification, and benign subtype classification, respectively. Ablation analysis showed that the complete framework improved F1-score by 2.52–4.71 percentage points over the visual-only hierarchical EfficientNet-B4 model. MorphoNet also achieved higher numerical performance than ResNet-50, DenseNet-121, and image-only EfficientNet-B4 baselines trained under the same protocol. The learned attention profiles were consistent with clinically relevant morphological characteristics. These findings demonstrate the complementary value of hierarchical classification, ABCD-guided feature fusion, and domain alignment for interpretable multi-source skin lesion analysis, although independent multicenter clinical validation remains necessary.

## 1. Introduction

Skin cancer is one of the most common malignancies worldwide, with its incidence continuing to increase because of genetic susceptibility and more exposure to ultraviolet radiation [1,2]. Although melanoma represents only a small proportion of all skin cancer cases, it accounts for the majority of skin cancer-related deaths due to its aggressive metastatic potential. Globally, approximately 132,000 new melanoma cases and 2–3 million non-melanoma skin cancer cases are diagnosed annually [3], and both the incidence and mortality of melanoma are projected to increase substantially by 2040 [4]. Early detection is therefore critical. The five-year survival rate exceeds 95% when melanoma is diagnosed at an early stage but falls to below 15% after the disease has progressed to advanced stages [5,6,7]. For example, the early screening program in Schleswig-Holstein, Germany, from 2003 to 2004 reduced roughly 50% of all melanoma-related deaths within the following five years [8], while a similar program at the Lawrence Livermore National Laboratory in the US (1984–1996) led to zero melanomic deaths [9]. These findings have driven continued interest in developing accurate and clinically interpretable computer-aided methods for skin lesion assessment [10].

In clinical practice, dermatologists commonly evaluate lesion morphology using well-established diagnostic criteria, including the ABCD rule, the seven-point checklist, and the Menzies method [11,12,13,14]. Although these criteria provide clinically meaningful guidance, their interpretation remains highly dependent on physician experience and may be influenced by lesion heterogeneity, resulting in observer variability in diagnosis. Recent advances in deep learning, particularly convolutional neural networks (CNNs), have achieved strong performance in automated dermoscopic image classification [15,16,17,18,19]. However, most CNN-based models function largely as black boxes, providing little explanation of the morphological features that support their predictions. This limited interpretability remains a major barrier to clinical acceptance, despite their high classification accuracy.

Another challenge is fine-grained lesion classification. Conventional single-stage models generally perform well in distinguishing benign from malignant lesions but often have difficulty separating visually similar lesion subtypes, especially when class distributions are highly imbalanced. In addition, combining images from multiple public datasets introduces domain shifts caused by differences in imaging devices, acquisition protocols, and patient populations, reducing model robustness across datasets. These limitations indicate the need for a classification strategy that reflects the hierarchical nature of clinical diagnosis, incorporates established dermatologic knowledge, and maintains reliable performance across heterogeneous data sources.

To address these challenges, we propose MorphoNet, a hierarchical diagnostic strategy that combines dermoscopic features with deep neural networks for automated skin lesion classification. The motivation comes from three observations: (1) single-stage models often struggle with the complexity of multi-class categorizations, especially in imbalanced datasets [20]; (2) integrating dermoscopic features with data-driven deep learning can enhance both accuracy and interpretability [19,21], and (3) the inherent complexity and limited interpretability of CNNs render them “black-box” models and make it difficult to understand how they reach a diagnosis based on medical images. The following are the main contributions of this study:

- A new two-stage identification CNN-based framework that first classifies lesions into benign or malignant, followed by subtype classification for benign and malignant lesions;
- A novel integration strategy that combines CNN-extracted features with quantitative dermoscopic features at multiple stages, which is compared and validated on benchmark datasets (HAM10000 and ISIC);
- Attention mechanisms learn interpretable feature weights for selected ABCD descriptors, thereby bridging the gap between established clinical assessment criteria and deep learning while improving the reliability, clinical relevance, and classification performance of the CAD framework.

The remaining parts of the paper are organized as follows: Section 2 discusses the relevant work related to the present research, Section 3 explains the collected data and detailed methodology, Section 4 provides results and discussions, and Section 5 presents the conclusions and important contributions of the work, as well as recommendations for future work and improvement.

## 2. Related Work

Computer-aided diagnosis (CAD) has been extensively applied in automated dermoscopic skin lesion analysis. Early CAD systems primarily relied on manually engineered descriptors, including shape, border, color, and texture features, which were combined with conventional machine learning classifiers such as support vector machines, decision trees, and k-nearest neighbors [19,20,21,22,23,24]. Other approaches used lesion segmentation, color analysis, clustering, and texture extraction to differentiate melanoma from benign nevi [25,26]. Although these approaches provided clinically interpretable representations, their performance depended heavily on manually designed features and segmentation quality, limiting their robustness to specific lesions and imaging conditions.

The emergence of deep learning has fundamentally transformed dermoscopic image analysis by enabling end-to-end feature learning directly from raw images. Early CNN-based studies combined deep convolutional features with traditional classifiers, transitioning past manual feature extraction [27,28,29]. The availability of large public datasets, including HAM10000 and the ISIC Archive, further accelerated the adoption of transfer learning and CNN-based classification models [30,31,32]. Numerous network architectures, including the VGG, ResNet, GoogLeNet, DenseNet, and EfficientNet, have demonstrated excellent performance in skin lesion classification, while ensemble learning strategies have further improved diagnostic accuracy by combining multiple models into better hybrid networks [33,34,35,36,37,38,39]. Prominent examples include the SkinDWNet, where a novel CNN architecture using depthwise dilated convolutions (DDCs) and feature reuse residual blocks (FRBs) was combined with gradient boosting (GB) techniques to create a highly accurate composite model [40], and the DSCIMABNet, where a multi-head attention depthwise separable CNN was ensembled with various modern networks trained on ImageNet to validate the hybridization strategy for deep learning-based skin lesion classification [41]. Despite these advances, many high-performing models remain computationally complex and function largely as “black boxes,” providing limited clinical interpretation in their diagnostic decisions.

To improve model transparency, recent studies have explored hybrid frameworks that integrate deep visual representations with clinically understandable information, including dermoscopic metadata and handcrafted morphological descriptors [19]. These approaches have demonstrated that incorporating prior clinical knowledge can enhance both diagnostic performance and interpretability. Nevertheless, several important challenges remain unresolved. First, most existing methods rely predominantly on deep visual features without explicitly incorporating established dermatological assessment criteria, such as the ABCD rule, into the decision-making process. Second, the majority of studies formulate skin lesion diagnosis as a single-stage multi-class classification problem, which is inconsistent with the hierarchical diagnostic workflow commonly followed in clinical practice. In many state-of-the-art models, including the aforementioned SkinDWNet and DSCIMABNet, architectures reflect these incongruous choices and could be improved with the addition of comprehensibility through quantitative measures and tiered classifications in skin lesion diagnoses, which would both better support real-world clinical evaluations. Finally, combining images from multiple public datasets introduces substantial domain shifts caused by differences in imaging devices, acquisition protocols, and patient populations, often reducing model robustness and generalizability.

The literature review shows that an interpretable hierarchical framework for skin lesion classification is necessary to address these limitations. MorphoNet integrates deep visual representations with quantitative ABCD morphological descriptors through an attention-guided feature fusion mechanism, enabling the model to learn clinically meaningful feature importance while preserving high diagnostic accuracy. Furthermore, CORAL-based domain alignment is incorporated to reduce feature-distribution discrepancies across datasets and improve robustness to heterogeneous dermoscopic datasets. Together, these components provide an interpretable and domain-robust framework for hierarchical skin lesion diagnosis.

## 3. Materials and Methods

### 3.1. Dataset Construction

**Figure 1:**
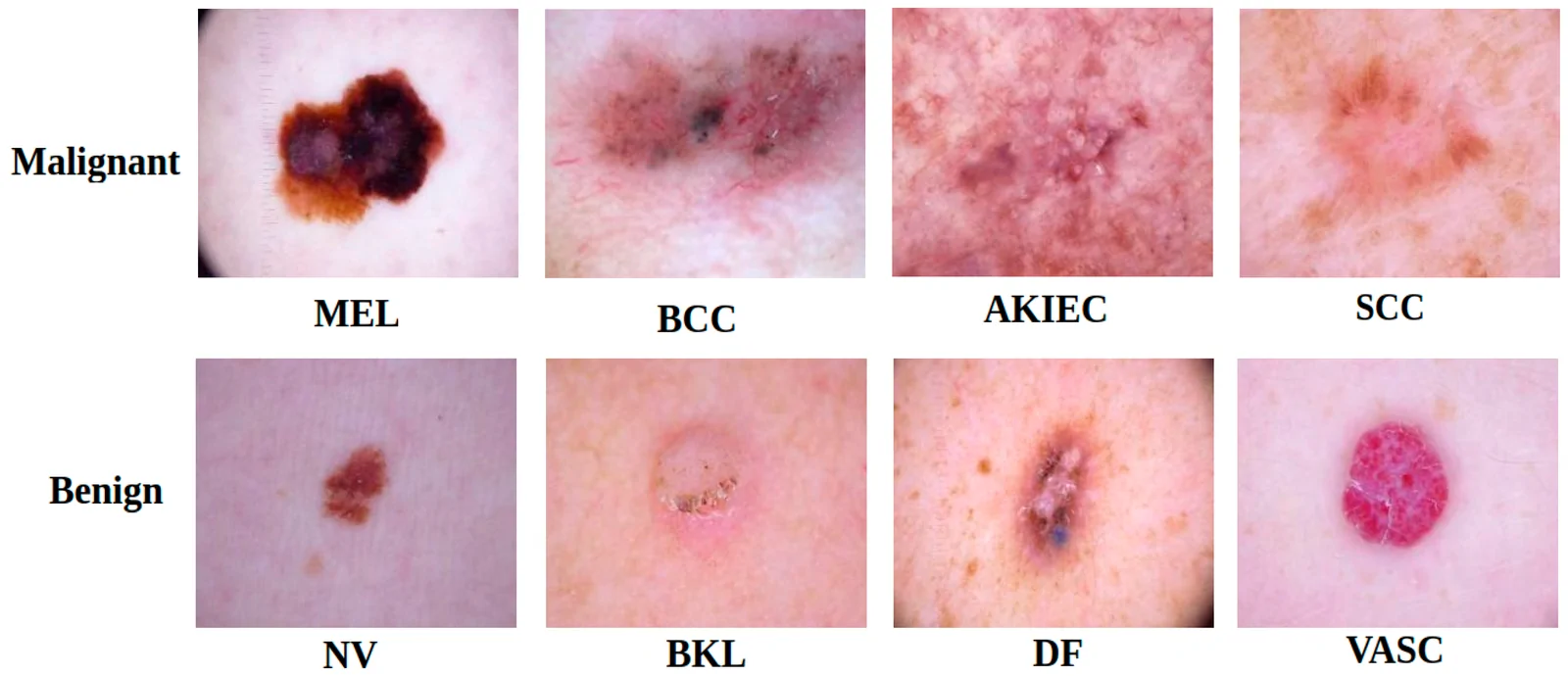
Representative dermoscopic images of malignant and benign lesion subtypes.

The dataset used in this study was constructed from three publicly available dermoscopic image datasets: HAM10000, ISIC 2019, and DERM12345. HAM10000 is a multi-source dermoscopic dataset containing 10,015 images from seven common pigmented lesion categories. DERM12345 is a multi-source dataset comprising 12,345 high-resolution dermoscopic images from 1627 patients and covering 40 lesion subclasses. In the present study, 503 DERM12345 images corresponding to the unified target categories were retained, including 266 SCC, 58 AKIEC, and 179 DF images. After dataset integration, a total of 16,845 dermoscopic images were included. The final dataset contained eight lesion categories: melanoma (MEL), basal cell carcinoma (BCC), actinic keratoses (AKIEC), squamous cell carcinoma (SCC), melanocytic nevi (NV), benign keratosis-like lesions (BKL), dermatofibroma (DF), and vascular lesions (VASC). The class-wise distribution of the integrated dataset across the three public sources is summarized in Table 1. For benign–malignant classification in Stage 1, NV, BKL, DF, and VASC were assigned to the benign group, while MEL, BCC, AKIEC, and SCC were assigned to the malignant group. Representative dermoscopic images of the eight lesion categories are shown in Figure 1.

Before model development, image identifiers were standardized across datasets, and duplicate records were removed. The integrated dataset was divided into training, validation, and test subsets at the patient level using a stratified 80:10:10 ratio. All images belonging to the same patient were assigned exclusively to one subset, thereby preventing patient-level overlap among the training, validation, and test sets. Stratification was performed according to lesion category as far as possible while preserving patient grouping. After splitting, patient identifiers and image identifiers were cross-checked across the three subsets, and no overlap was detected. Data augmentation, weighted sampling, and other class-balancing procedures were applied only to the training set, whereas the validation and test sets retained their original distributions.

To improve representation consistency across the integrated multi-source dataset, CORAL domain alignment was introduced during training. This regularization encourages the model to learn source-invariant feature representations while preserving diagnostic information for lesion classification.

### 3.2. Development of MorphoNet Model

**Figure 2:**
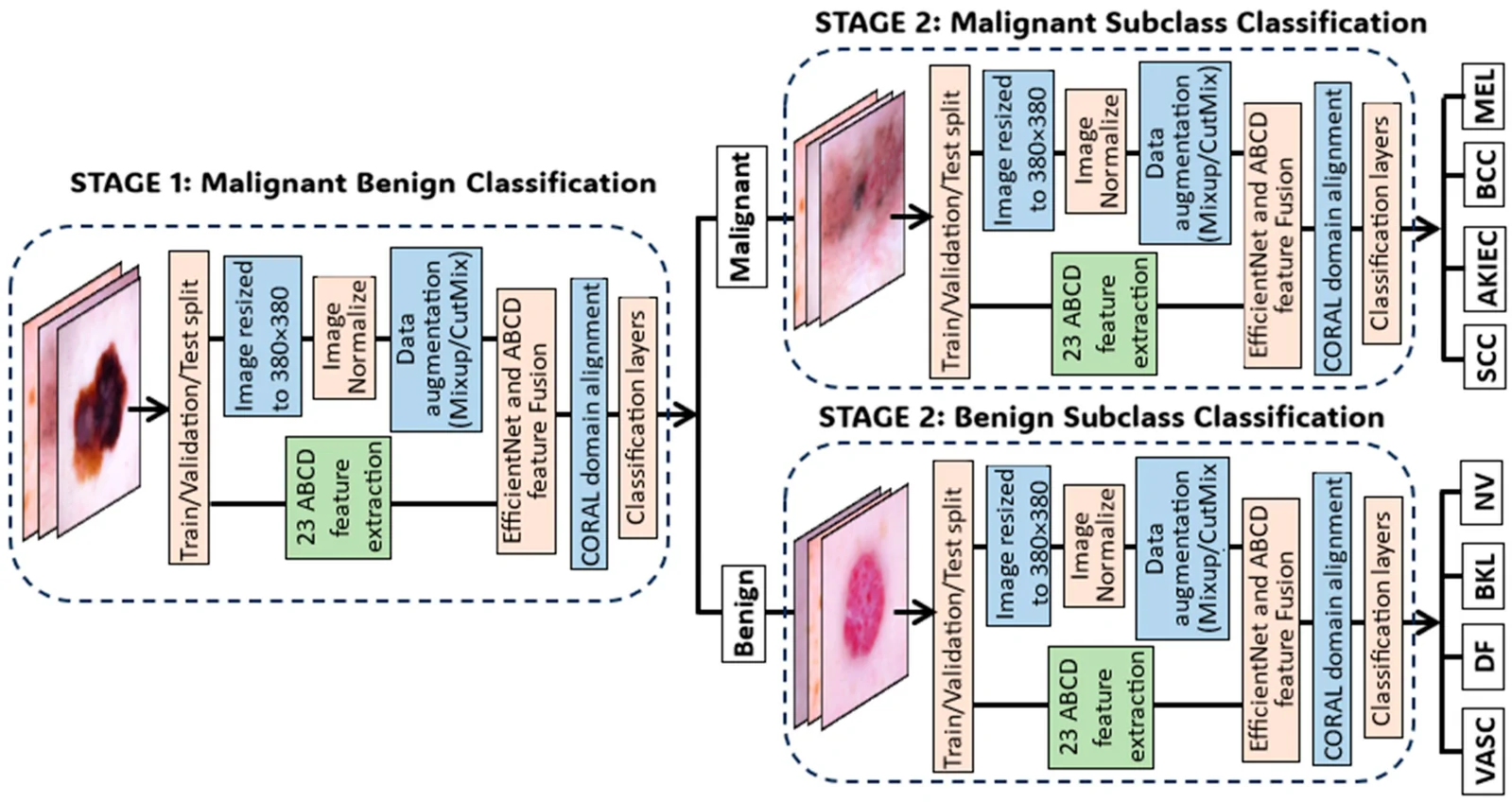
Architecture of the proposed MorphoNet model.

The proposed MorphoNet framework is shown in Figure 2. MorphoNet is a two-stage EfficientNet-B4-based model that combines CNN-extracted features with quantitative ABCD dermoscopic descriptors, including asymmetry, border irregularity, color variance, and diameter-related features. In Stage 1, the model performs benign–malignant classification. In Stage 2, two specialized branches further classify malignant lesions into MEL, BCC, AKIEC, and SCC, and benign lesions into NV, BKL, DF, and VASC. An attention mechanism learns specific weights for the ABCD descriptors, and the weighted morphological features are fused with EfficientNet-B4 visual features before classification.

#### 3.2.1. Data Preprocessing and Augmentation

Data preprocessing was applied to improve input consistency, reduce image-level noise, and emphasize regions containing diagnostically relevant lesion details. Together with augmentation and class balancing, preprocessing could reduce overfitting and improve model stability [42,43,44].

Before training the MorphoNet model, the input images underwent the following preprocessing methods:

Resizing: The images were resized to 380 by 380 pixels, the input dimensions required for EfficientNet-B4.

Rescale: Pixel values were converted to PyTorch format and rescaled to the range [0, 1] for numerical stability.

Normalization: Pixel values were normalized using the channel-wise mean and standard deviation.

After preprocessing, two data augmentation techniques, MixUp and CutMix, were employed within the training set to improve generalization and mitigate overfitting. Validation and test images were not augmented. Class-balancing strategies were applied after data splitting and only within the training set. Class imbalance was addressed using inverse-frequency weighted random sampling within the training set, which increased the sampling probability of underrepresented lesion categories. Validation and test samples were not resampled, and standard cross-entropy loss was used for model optimization. MixUp performs global linear interpolation to improve generalization, whereas CutMix maintains spatial structure through localized region replacement and provides strong regularization. MixUp constructs new training samples through random combinations of image-label pairs [45]. Given the batch of image ${\left\{X_{i}\right\}}_{i=1}^{N}$ and its corresponding labels ${\left\{Y_{i}\right\}}_{i=1}^{N}$, the MixUp algorithm generates augmented samples as $\tilde{X_{i}}=\lambda \cdot X_{i}+(1-\lambda )\cdot X_{\pi (i)}$ and $\tilde{Y_{i}}=\lambda \cdot Y_{i}+(1-\lambda )\cdot Y_{\pi (i)}$, where π is the random permutation function among samples, and λ is sampled from the Beta distribution β(0.2, 0.2).

For CutMix data augmentation, two random training images were selected, and a random patch was cut from one image and pasted into the other, with the mixed label determined by the area proportions in the final image [46]. CutMix was stochastically applied with 20% probability during training iterations and was not applied to validation or test images.

#### 3.2.2. Feature Fusion Between EfficientNet Features and ABCD Features

To incorporate clinically meaningful dermoscopic information into the deep learning pipeline, each image was processed by both a CNN branch and an ABCD feature branch. The CNN branch extracted visual information from EfficientNet-B4, whereas the ABCD branch extracted features specific to the ABCD rule (Table 2). Different ABCD features were selected at each stage according to the corresponding feature extractor. The attention mechanism was configured accordingly.

Asymmetrical shape is one of the important indicators for cancerous skin [19]. In this research, asymmetrical features were found using a grayscale image and Otsu’s thresholding. The binary mask of the largest contour was calculated, as well as its area, perimeter, and center coordinates, using a contour approximation and polygon filling algorithm. The lesion was then divided into left, right, top, and bottom halves, and the horizontal and vertical asymmetry features were calculated as normalized absolute differences between the sums of each respective region. The asymmetry ratio between horizontal and vertical quantifies the directional preference of asymmetry, while the asymmetry entropy measures the uncertainty in the overall asymmetrical distribution of the skin lesion.

Border irregularity is a critical diagnostic criterion for melanoma detection. The perimeter-to-area ratio measures the roughness relative to the lesion size, while boundary complexity compares the convex hull perimeter to the actual perimeter to detect jagged edges. Circularity and compactness evaluate the shape regularity by calculating how close the lesion shape is to a perfect circle and how efficiently its area is enclosed within its perimeter. Fractal dimension captures the self-similarity of the border, with higher values indicating more irregular boundaries and lower values indicating regularity.

Color features in this research comprehensively analyze color variation and distribution among both RGB (red, green, and blue) and HSV (hue, saturation, and brightness) color spaces to detect the irregular pigmentation of skin lesions. The color entropy value was further computed for each RGB channel, while a diversity index assessed the overall color heterogeneity by averaging the variance across the RGB channels.

The diameter component was inspired by the clinical D criterion of the ABCD rule. In the implementation, diameter-related features were calculated in image space. The lesion contour was fitted with an ellipse, and the major-axis length was measured in pixels and normalized by the diagonal length of the corresponding image. Therefore, these descriptors quantify relative lesion extent and do not represent an absolute clinical measurement in millimeters.

**Figure 3:**
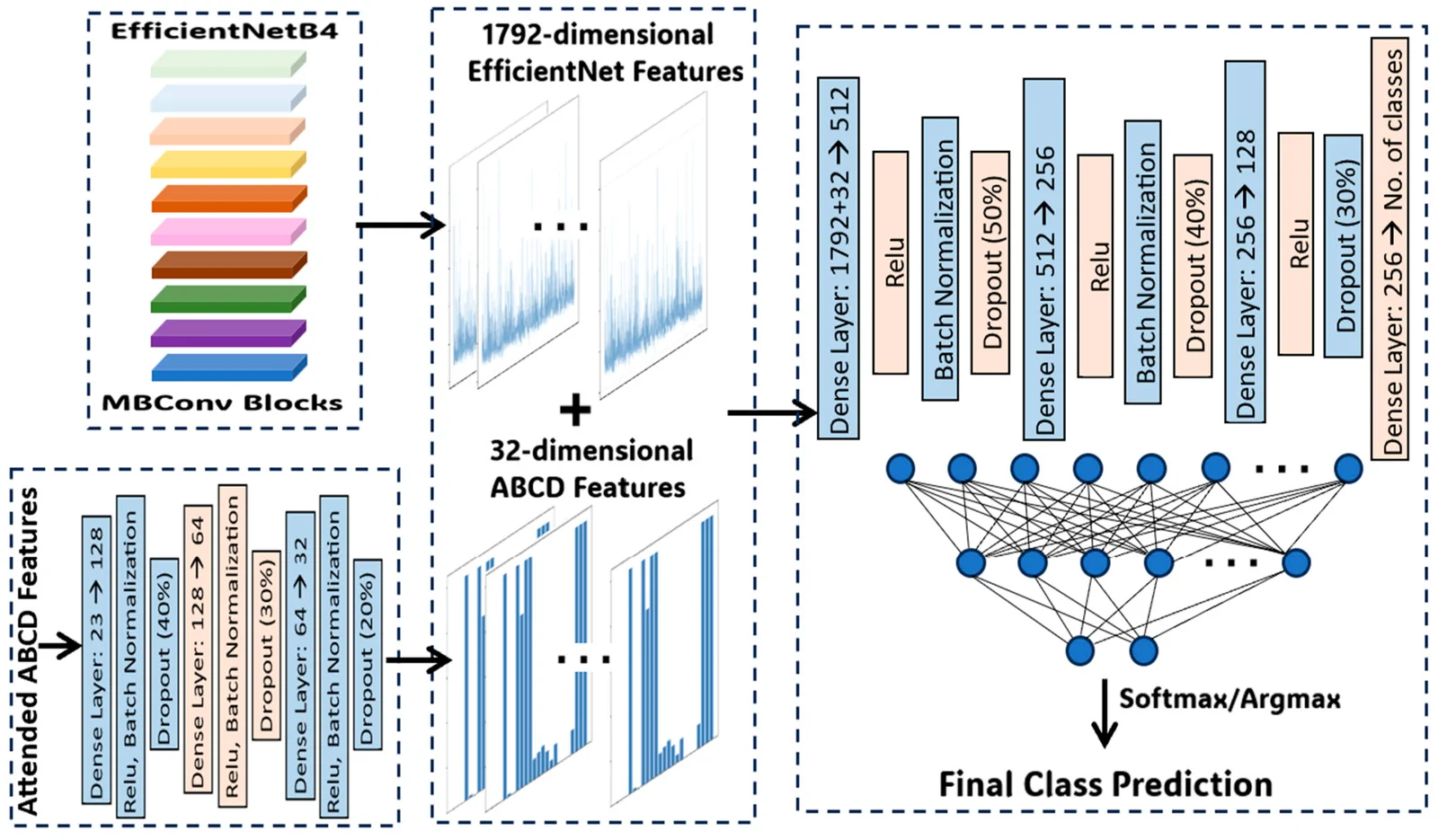
Feature fusion and class prediction in the Stage 1 model using EfficientNetB4.

MorphoNet implements a dual-branch neural network that separately processes visual features through EfficientNet-B4 and dermoscopic descriptors through the ABCD feature branch (Figure 3). EfficientNet-B4 generates a high-dimensional visual embedding that captures lesion morphology, color, and texture patterns from the input image. In parallel, the ABCD branch produces a vector of stage-specific quantitative dermoscopic features. The attention module learns feature-wise weights for the ABCD vector, producing weighted ABCD features that emphasize diagnostically informative descriptors. The weighted ABCD representation is then fused with the EfficientNet-B4 visual embedding and passed through fully connected classification layers with normalization and dropout. The same fusion principle is applied to Stage 1 and both Stage 2 subtype classifiers, while each stage learns its own task-specific ABCD attention weights.

#### 3.2.3. Attention Mechanism for ABCD Feature Weights

To better interpret the model results, an attention mechanism was deployed to learn which of the ABCD features are diagnostically important. Given all ABCD features f, the attention weights are computed through a two-layer network:

(1) $$w_{f}=\sigma \left(w_{2}\times tanh\left(w_{1}\times f+b_{1}\right)+b_{2}\right),$$

(2) $${\hat{w}}_{f}=23\times \frac{w_{f}}{\sum_{i=1}^{23}w_{f}},$$

where $w_{1}$ and $w_{2}$ are learnable weight matrices, $b_{1}$ and $b_{2}$ are bias vectors, and σ is the sigmoid function ensuring the $w_{f}$ is within ${\left[0,1\right]}^{23}$ for ABCD features. The weights $w_{f}$ were normalized by their sum to preserve their relative importance, and the attended features $f^{\prime }$ were obtained through element-wise multiplication:

(3) $$f^{\prime }=f⊙{\hat{w}}_{f},$$

where ⊙ is the Hadamard product. Values near 1 in $w_{f}$ indicate important features while values near 0 indicate irrelevant features.

At the start of training, the weight matrices ($w_{1}$ and $w_{s}$) were randomly initialized using Kaiming initialization, and bias vector ($b_{1}$) was initialized as zeros. During each training iteration, the attention weights $w_{f}$ were calculated in the forward pass by passing input features through learned weight matrices, and then the weight matrices were updated through backpropagation to minimize classification loss, causing the model to automatically learn the attention weights of discriminative features to improve prediction accuracy.

#### 3.2.4. CORAL Domain Alignment

Although HAM10000, ISIC 2019, and DERM12345 were jointly integrated into the training dataset, these public dermoscopic image sources may still differ in imaging conditions, acquisition devices, color distribution, image resolution, and class composition. Such inter-dataset heterogeneity may cause the model to learn dataset-specific feature patterns rather than lesion-related diagnostic features. To reduce this dataset-source bias, CORAL regularization was introduced at the feature embedding level. CORAL encourages feature representations from different data sources to share similar second-order statistics by minimizing the distance between their covariance matrices.

The total training loss was defined as $L_{total}=L_{cls}+\lambda L_{CORAL}$, where $L_{cls}$ denotes the classification loss, $L_{CORAL}$ denotes the CORAL regularization loss, and λ controls the contribution of domain alignment. The CORAL weight was selected using the validation set from candidate values of 0.01, 0.05, 0.1, and 0.2. A value of (λ = 0.1) provided the best overall validation macro-F1 across the three classification tasks and was therefore fixed for all final experiments. The test set was not used during this selection process.

#### 3.2.5. Ablation Study Design

To quantify the individual contributions of the ABCD-guided feature fusion module and CORAL-based domain alignment, a component-wise ablation study was conducted using three model configurations. All configurations retained the same two-stage hierarchical classification strategy, EfficientNet-B4 backbone, data partitions, preprocessing procedures, augmentation strategies, class-balancing method, optimization settings, and model selection criteria. Therefore, differences in performance could be attributed primarily to the inclusion of the ABCD and CORAL components rather than to changes in the backbone architecture or experimental protocol.

The first configuration, referred to as the visual-only hierarchical baseline, used only the image features extracted by EfficientNet-B4 and excluded both the ABCD feature branch and CORAL regularization.

The second configuration, denoted as EfficientNet-B4 + ABCD, incorporated the quantitative ABCD descriptors through the same attention-guided feature fusion module used in MorphoNet, but CORAL regularization was disabled by setting its loss weight to zero. This configuration was used to isolate the contribution of clinically informed morphological feature fusion.

The third configuration was the complete MorphoNet model, which combined EfficientNet-B4 visual representations, attention-weighted ABCD descriptors, and CORAL-based domain alignment. The CORAL weight was set to 0.1, consistent with the final model configuration.

Each configuration was independently trained for Stage 1 benign–malignant screening, Stage 2 malignant subtype classification, and Stage 2 benign subtype classification. All experiments were repeated using the same three random seeds. Model selection was based exclusively on validation-set performance, and the sealed test set was evaluated only after the final model checkpoint had been selected. Accuracy, precision, recall, and F1-score were reported as the mean ± standard deviation across the three runs.

#### 3.2.6. Baseline Models and Comparison Protocol

MorphoNet was compared with three representative image-based convolutional neural networks: ResNet-50, DenseNet-121, and EfficientNet-B4. Each backbone was adapted to the same two-stage hierarchical protocol and was trained separately for benign–malignant screening, malignant subtype classification, and benign subtype classification. The baseline models used dermoscopic images only and did not include the ABCD feature branch, attention-guided feature fusion, or CORAL regularization.

All models were evaluated using identical dataset partitions, preprocessing and augmentation procedures, class-balancing strategies, optimization settings, and random seeds. Accuracy, precision, recall, and F1-score are reported as the mean ± standard deviation across three runs.

#### 3.2.7. Accuracy Assessment

To evaluate MorphoNet, the integrated dataset was divided into stratified training, validation, and test subsets using a standard proportional split. Model selection and hyperparameter adjustment were performed without using the sealed test set. The final test set was evaluated only after the model configuration was fixed. Performance was assessed using accuracy, precision, recall, and F1-score. Confusion matrices were additionally generated to visualize class-wise classification performance. All final experiments were repeated using three random seeds, and the four evaluation metrics are reported as the mean ± standard deviation across the three runs.

Accuracy measures the proportion of correctly classified images among all evaluated samples. Precision, recall, and F1-score were calculated for each class to characterize false-positive and false-negative behavior, which is particularly important for rare malignant categories.

(4) $$Accuracy=\frac{TP+TN}{TP+TN+FP+FN},$$

(5) $$Precision=\frac{TP}{TP+FP},$$

(6) $$Recall=\frac{TP}{TP+FN},$$

(7) $$F1=\frac{2\times Precision\times Recall}{Precision+Recall}.$$

Confusion matrices were generated for Stage 1 benign–malignant classification, Stage 2 malignant subtype classification, and Stage 2 benign subtype classification to visualize class-wise misclassification patterns.

Fine-tuning was performed using the AdamW optimizer with an initial learning rate of 1 × 10^−4^, a batch size of 16, and a weight decay of 1 × 10^−4^. The learning rate was reduced to 1 × 10^−6^ using cosine annealing over 30 epochs. All models were implemented using PyTorch version 2.1.0 and trained with CUDA acceleration.

## 4. Experimental Results and Discussion

The training, validation, and final evaluation of MorphoNet are presented in this section. Because the final framework integrates EfficientNet-B4 visual features, ABCD feature attention, and CORAL domain alignment, the results focus on convergence behavior, attention-based interpretability, and the final Stage 1 and Stage 2 classification performance.

### 4.1. Ablation Study

A component-wise ablation study was conducted to evaluate the contributions of ABCD-guided feature fusion and CORAL-based domain alignment. Three configurations were compared under identical data splits and training settings: a visual-only hierarchical EfficientNet-B4 model, EfficientNet-B4 with ABCD attention, and the complete MorphoNet model with both ABCD attention and CORAL. The results are summarized in Table 3.

Adding ABCD features consistently improved performance across all three tasks. Accuracy increased from 91.84% to 93.08% for benign–malignant screening, from 85.73% to 87.58% for malignant subtype classification, and from 88.93% to 90.47% for benign subtype classification. The corresponding F1-score improvements were 1.47, 2.32, and 2.27 percentage points.

CORAL regularization provided further gains, resulting in final accuracies of 94.01%, 89.05%, and 91.84%, with F1-scores of 93.70%, 84.00%, and 87.97%, respectively. Compared with the visual-only baseline, the complete MorphoNet model improved accuracy by 2.17–3.32 percentage points and F1-score by 2.52–4.71 percentage points. These results indicate that ABCD-guided feature fusion and CORAL regularization make complementary contributions, particularly for fine-grained subtype classification.

### 4.2. Comparison with Baseline Models

MorphoNet was compared with ResNet-50, DenseNet-121, and EfficientNet-B4 under identical experimental conditions. As shown in Table 4, EfficientNet-B4 achieved the strongest performance among the image-only baselines across all three classification tasks. DenseNet-121 consistently outperformed ResNet-50 but remained below EfficientNet-B4.

Compared with the image-only EfficientNet-B4 baseline, MorphoNet improved accuracy by 2.17, 3.32, and 2.91 percentage points for benign–malignant screening, malignant subtype classification, and benign subtype classification, respectively. The corresponding F1-score improvements were 2.52, 4.48, and 4.71 percentage points. The larger gains in the two subtype-classification tasks indicate that the integration of ABCD morphological descriptors and CORAL-based domain alignment was particularly beneficial for fine-grained and class-imbalanced lesion recognition.

These results demonstrate that the performance of MorphoNet cannot be attributed solely to the EfficientNet-B4 backbone. Instead, the clinically guided feature-fusion and domain-alignment components provided complementary information beyond image-based CNN representations.

### 4.3. Attention Weights for ABCD Features

In this study, attention weights were analyzed to evaluate the relative contribution of individual ABCD features to each classification task. These weights provide interpretability signals within the model and indicate which morphological features contributed more strongly to the fused representation. They should therefore be interpreted as model-specific explanatory indicators rather than as independent clinical evidence.

**Figure 4:**
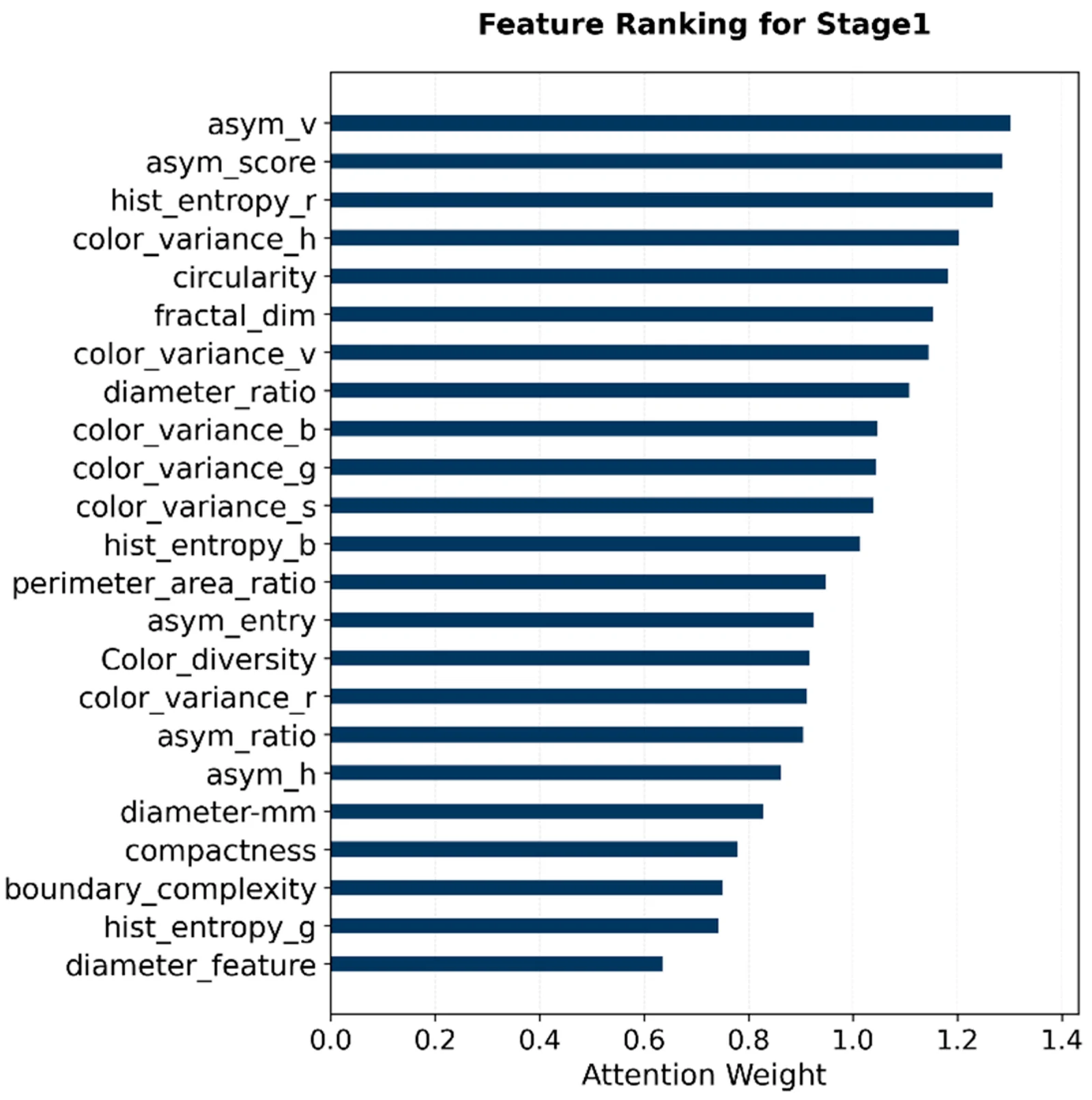
Attention weights of ABCD features in Stage 1 for malignant vs. benign classification.

For Stage 1 benign–malignant classification, the attention profile showed that asymmetry-related features (asym_v and asym_score) and color heterogeneity (hist_entropy_r and color_variance_h) descriptors received relatively high weights (Figure 4). This pattern suggests that global morphological imbalance and pigmentation variability provide important discriminative information for separating malignant from benign lesions.

**Figure 5:**
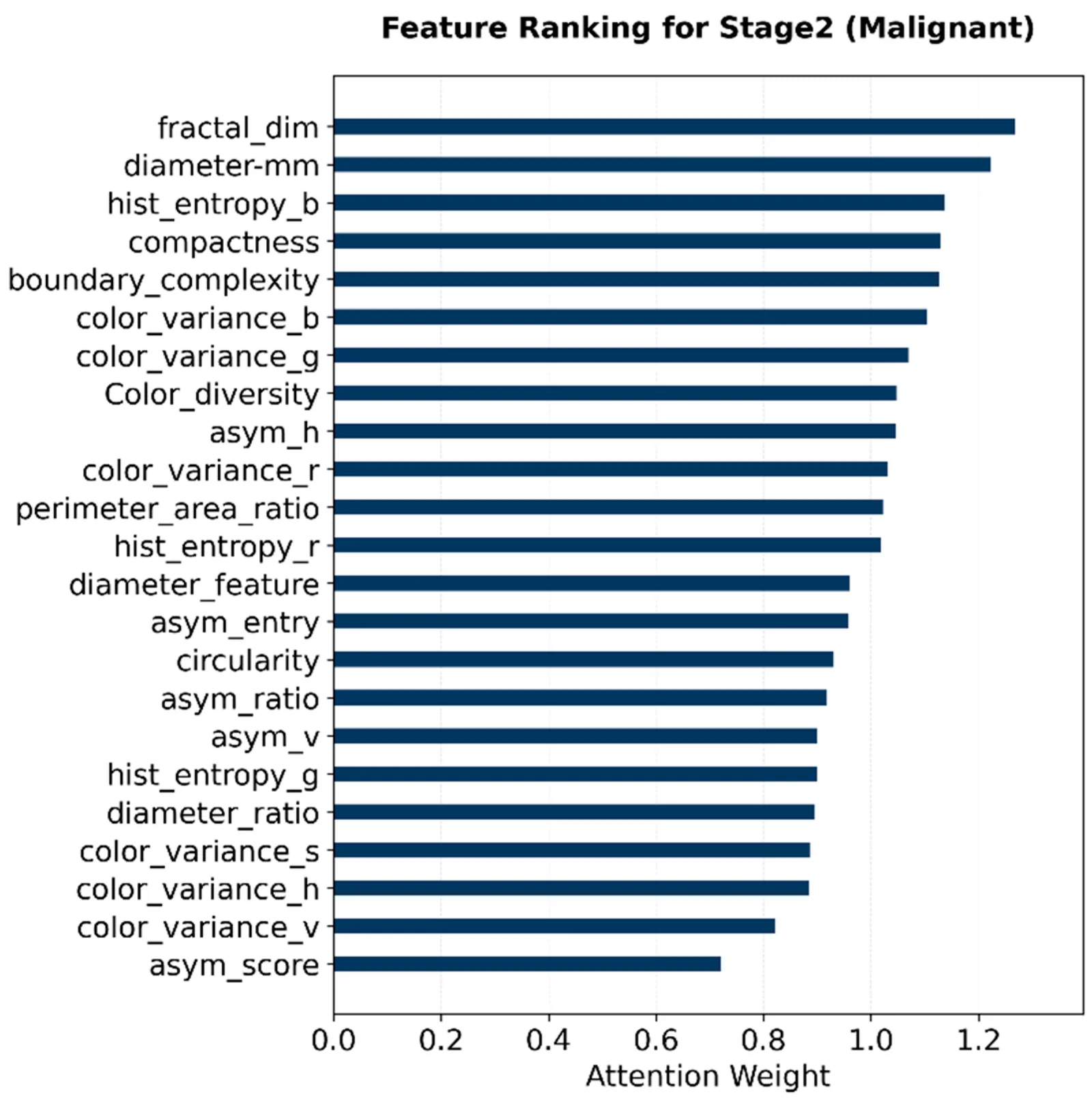
Attention weights of ABCD features in Stage 2 for malignant-type classification.

For Stage 2 malignant subtype classification, the highest-weighted descriptors were mainly related to border complexity, compactness, fractal dimension, diameter-related measurements, and color entropy (Figure 5). This attention pattern suggests that the malignant branch relied more strongly on lesion boundary irregularity, shape complexity, and color heterogeneity when distinguishing MEL, BCC, AKIEC, and SCC. These weights indicate that when classifying specific malignancy types, the model relies primarily on shape complexity and boundary irregularity.

**Figure 6:**
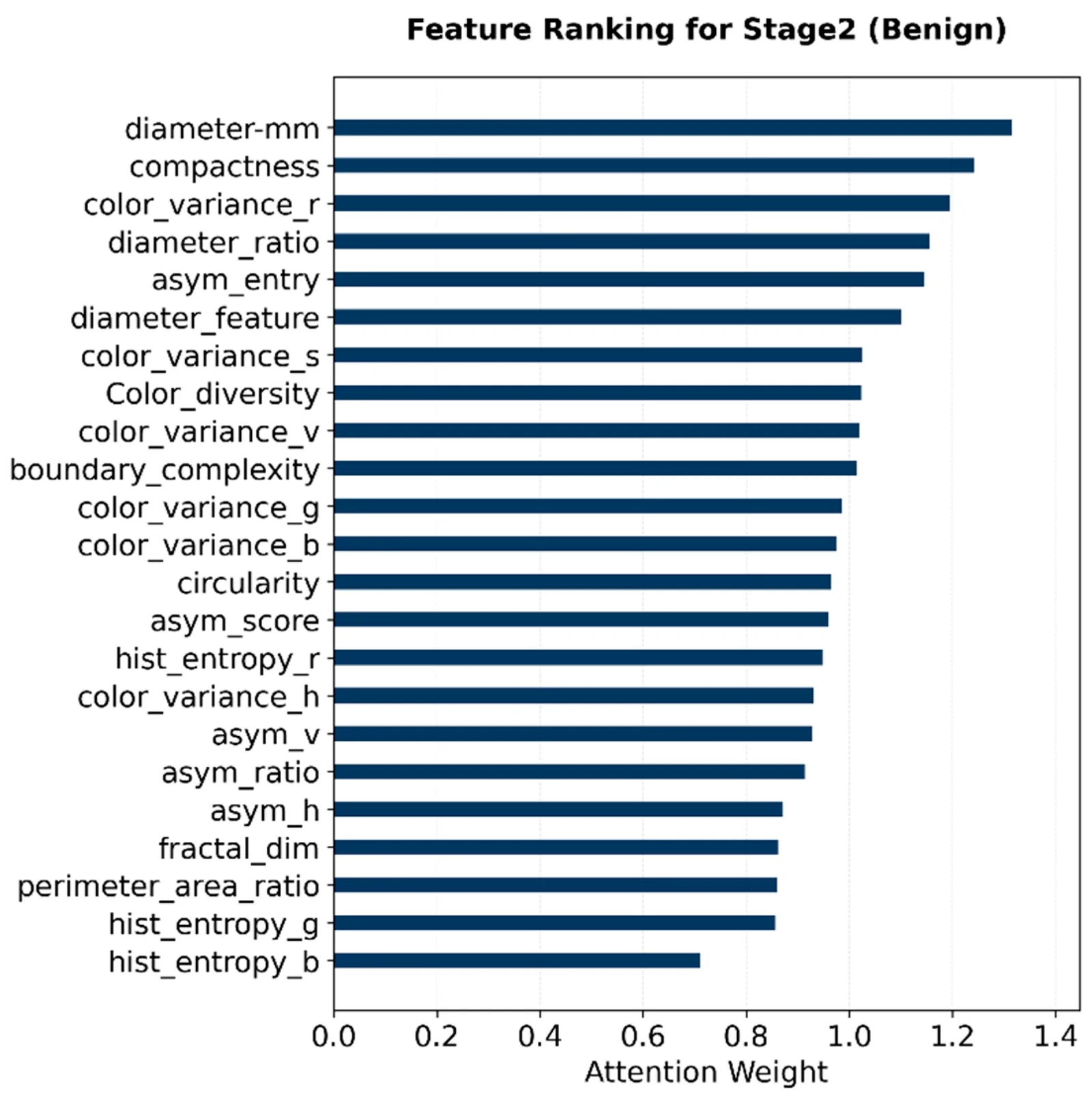
Attention weights of ABCD features in Stage 2 for benign-type classification.

For Stage 2 benign subtype classification, diameter-related features, compactness, and selected color-variation descriptors received relatively high attention weights (Figure 6). These findings indicate that lesion size, shape regularity, and color distribution contributed to the discrimination of NV, BKL, DF, and VASC within the benign branch.

Overall, the learned attention patterns differed across the three classification tasks. Stage 1 emphasized broad asymmetry and pigmentation heterogeneity for benign–malignant screening, whereas the Stage 2 malignant branch assigned greater importance to border and texture complexity. In contrast, the Stage 2 benign branch placed more emphasis on size and regularity descriptors. These findings support the use of ABCD-guided attention as a clinically interpretable component of MorphoNet.

### 4.4. Model Training and Validation Accuracy and Loss

**Figure 7:**
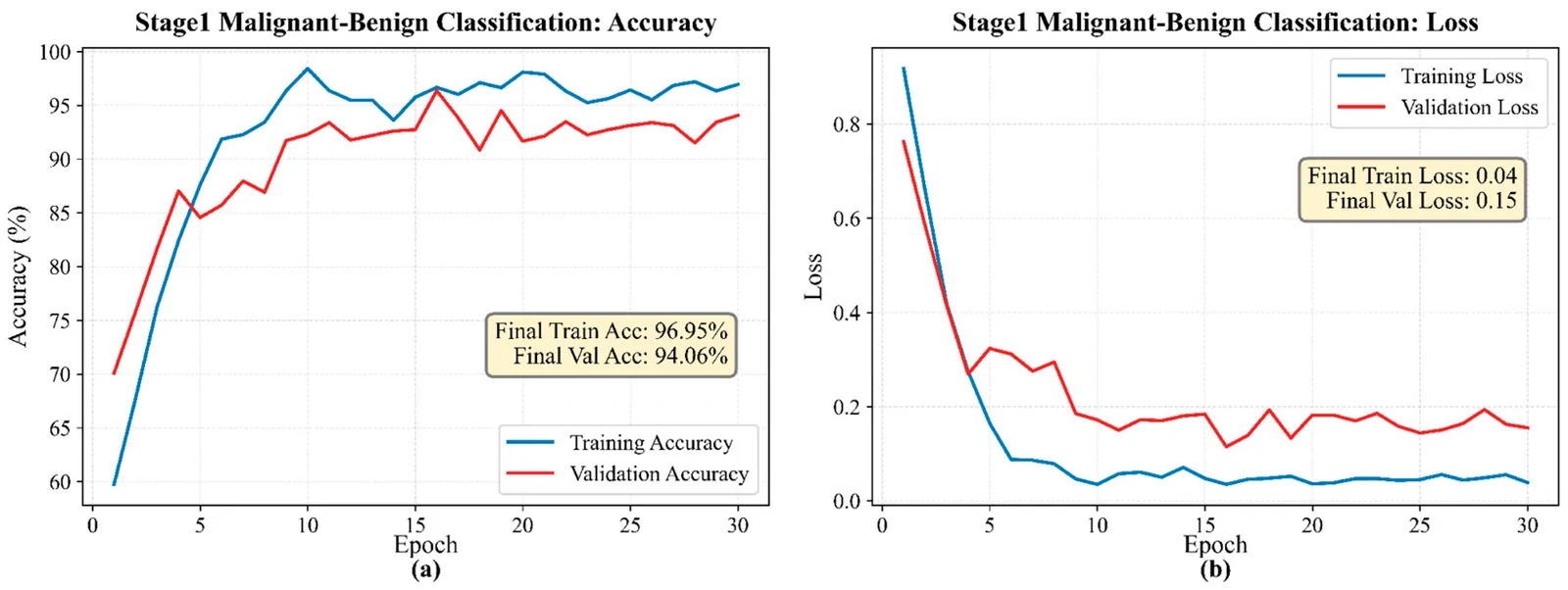

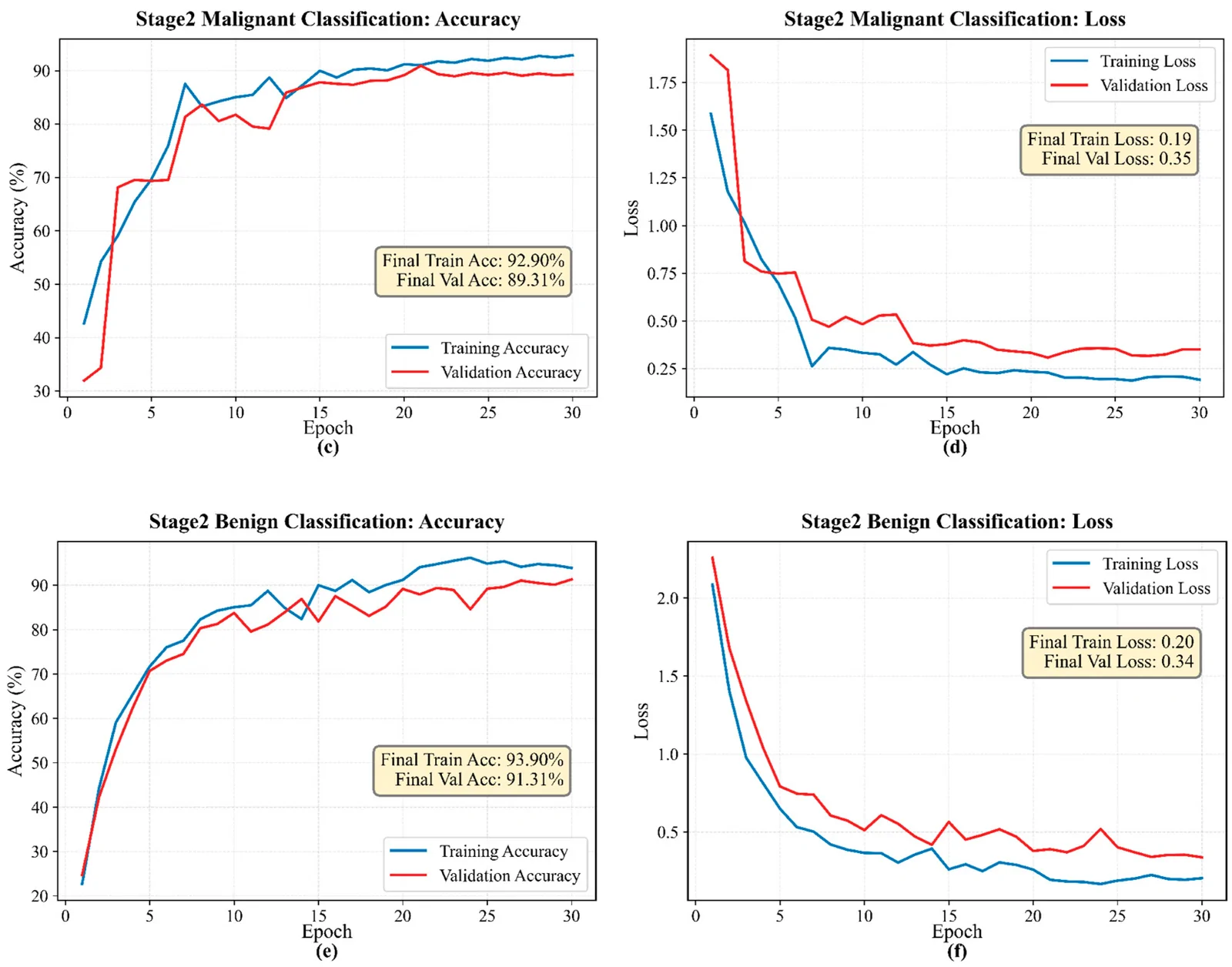
Training and validation accuracy and loss curves of MorphoNet: (**a**) accuracy curves on Stage 1 malignant-benign classification, (**b**) loss curves on Stage 1 malignant-benign classification, (**c**) accuracy curves on Stage 2 malignant classification, (**d**) loss curves on Stage 2 malignant classification, (**e**) accuracy curves on Stage 2 benign classification, and (**f**) loss curves on Stage 2 benign classification.

Training and validation curves were used to evaluate the convergence behavior of MorphoNet across Stage 1 and the two Stage 2 subtype branches. Across the integrated multi-source dataset, CORAL regularization was used to improve the consistency of feature representations across datasets during training. In Figure 7a, the accuracies of both training and validation increase rapidly at the beginning of training for the first 10 epochs and then gradually converge and remain stable for the other twenty epochs, showing a generally steady training and validation process. The small gap between the training accuracy and validation accuracy indicates good generalization in classifying malignant and benign. In the loss curve for the Stage 1 binary model (Figure 7b), the training loss exhibits a stable downward trend that gradually converges to approximately 0.05 near the end of training, while the validation loss converges to approximately 0.15. Both losses remain lower than those of the other two models. The Stage 1 classifier showed the most stable convergence, which is consistent with the relatively simpler binary classification task. The contrast between the loss and accuracy curves shows that while the Stage 1 binary model had high variability in its confidence in predictions, its classifications remained generally accurate throughout.

For the Stage 2 malignant classification model, both the loss and accuracy curves are relatively steady and convergent throughout the training process (Figure 7c,d). In the accuracy curve, both the training and validation accuracies increased sharply in the first 15 epochs (from 40% to 85%) and gradually leveled out for the remainder of the training and validation process. In the loss curve, the values stabilize at roughly the 20th epoch and converge to around 0.20 for the training loss and 0.35 for the validation loss. Compared with the Stage 1 model, the Stage 2 malignant branch exhibited higher variability during validation, likely because AKIEC and SCC had smaller sample sizes and shared visual similarities with other malignant subtypes.

The Stage 2 benign classification model displays moderately similar trends in the loss curve, with the training loss and validation loss undergoing rapid decreases in the first 5 to 10 epochs and stabilizing afterward (Figure 7e,f). The benign model achieved accuracies similar to those of the malignant model, with approximately 3–4 percentage points lower than those achieved by the first-stage binary classifier, which could also be caused by the dominance of NV and the limited number of DF and VASC samples.

Overall, the training accuracy increased during the early epochs and gradually stabilized, while the training loss decreased and approached a plateau. All the accuracy and loss curves are smooth and stable with rapid convergence and consistently high accuracy and low losses. Although slight overfitting could still be found between the training-validation gaps, the overall learning behavior supports the reliability and practical use of this two-stage classification framework. The validation curves showed similar trends, indicating that the model reached stable convergence without obvious divergence between training and validation performance. The difference in loss suggests mild overfitting, but taking into consideration the low and consistent values of validation loss, the model should still generalize well to unseen data.

### 4.5. Classification Performance

The final classification performance of MorphoNet was evaluated separately for Stage 1 benign–malignant screening, Stage 2 malignant subtype classification, and Stage 2 benign subtype classification. Performance was evaluated using accuracy, precision, recall, and F1-score, while confusion matrices were used to visualize class-wise misclassification patterns. Final test results across three random seeds are reported as the mean ± standard deviation.

**Figure 8:**
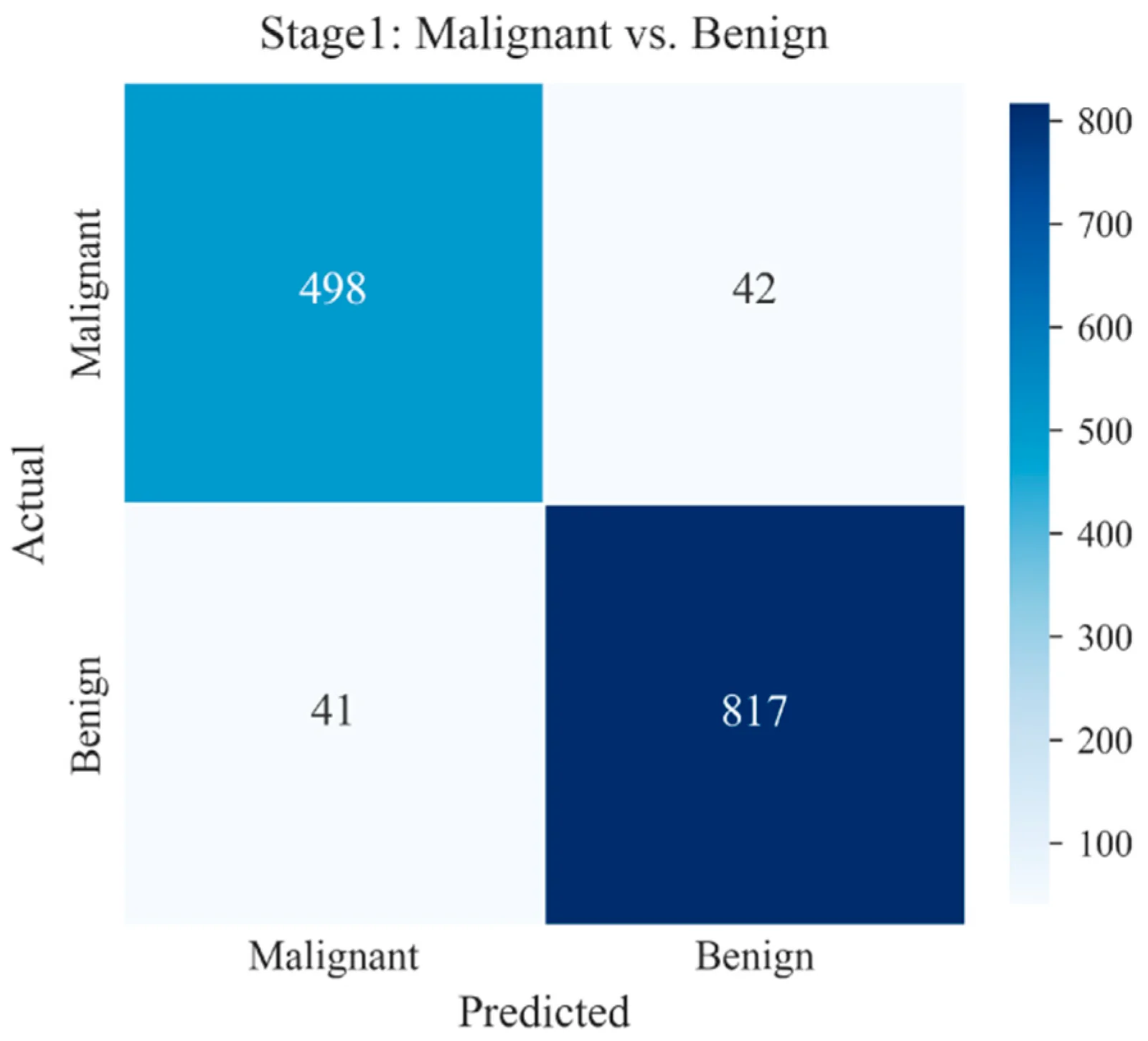
Representative test-set confusion matrix of the Stage 1 benign–malignant classifier.

For Stage 1, MorphoNet achieved 94.01 ± 0.50% accuracy, 93.73 ± 0.56% precision, 93.68 ± 0.55% recall, and 93.70 ± 0.55% F1-score (Table 3). High precision and recall in both the malignant class (92%/95%) and the benign class (92%/95%) indicate that the Stage 1 model minimizes false positives for malignant cases and false negatives for benign cases. The detailed class-wise performance metrics of the Stage 1 classifier are summarized in Table 5. The corresponding confusion matrix (Figure 8) showed the distribution of correctly and incorrectly classified benign and malignant lesions. The numbers of false-negative and false-positive predictions were similar to those of the malignant class, exhibiting a slightly higher misclassification rate. Therefore, the model is highly reliable when labeling true malignancies while simultaneously missing very few benign cases.

**Figure 9:**
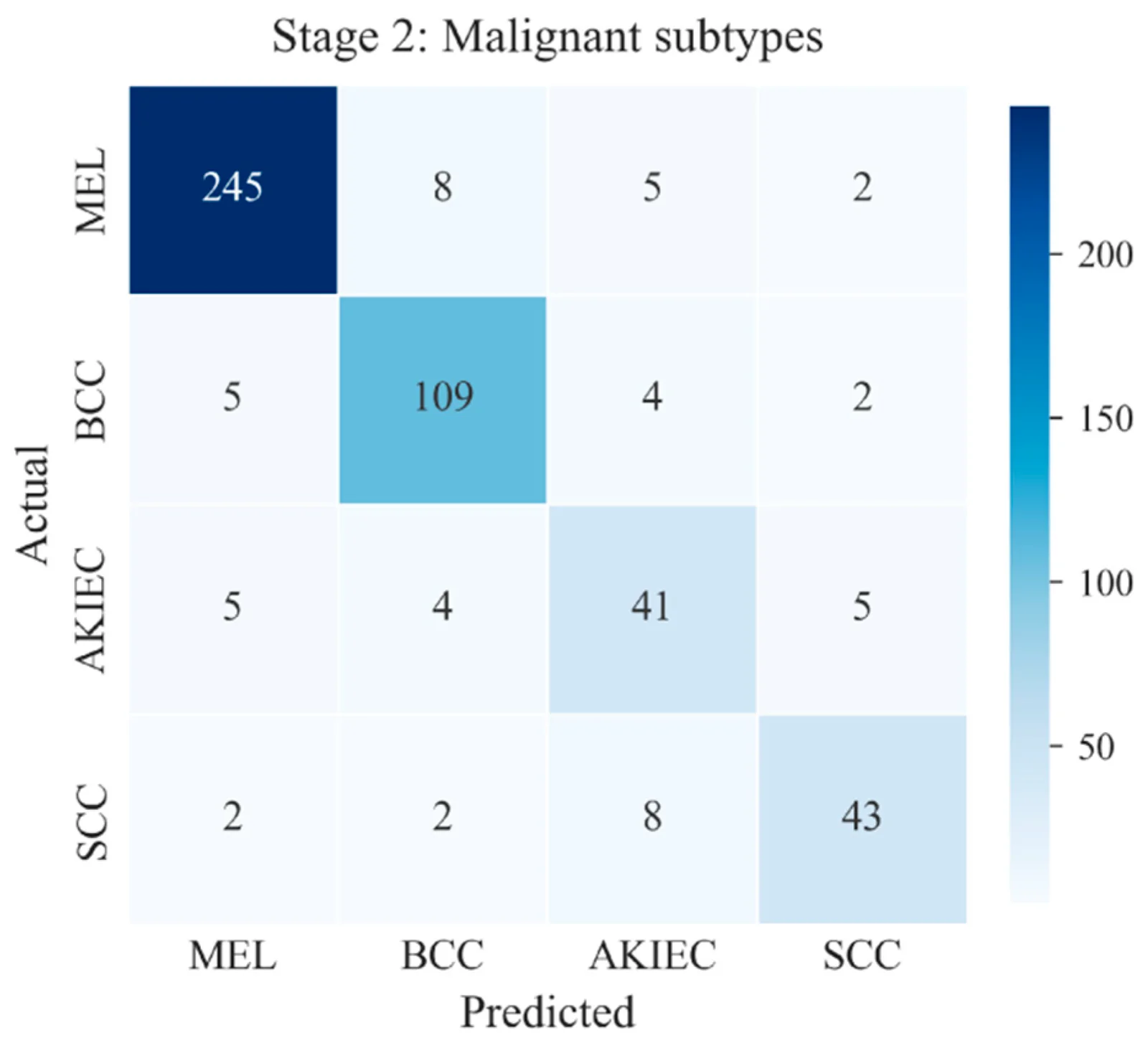
Representative test-set confusion matrix of the Stage 2 malignant subtype classifier.

Among the four malignant subtypes, MEL achieved the best precision (94.39%), recall (94.15%), and F1-score (94.27%), and all four classes achieved high accuracies (93.93–95.27%). The detailed classification performance of each malignant subtype is summarized in Table 6. Due to the smaller sample size, the AKIEC and SCC exhibit relatively low precision (70.68% and 82.69%, respectively) and recall (74.53% and 78.20%), demonstrating that the model is still highly effective at detecting AKIEC and SCC but sometimes classifies non-AKIEC or SCC lesions into the two categories. The corresponding confusion matrix of the Stage-2 malignant subtype classifier is shown in Figure 9, illustrating the class-wise prediction distribution among the four malignant subtypes.

**Figure 10:**
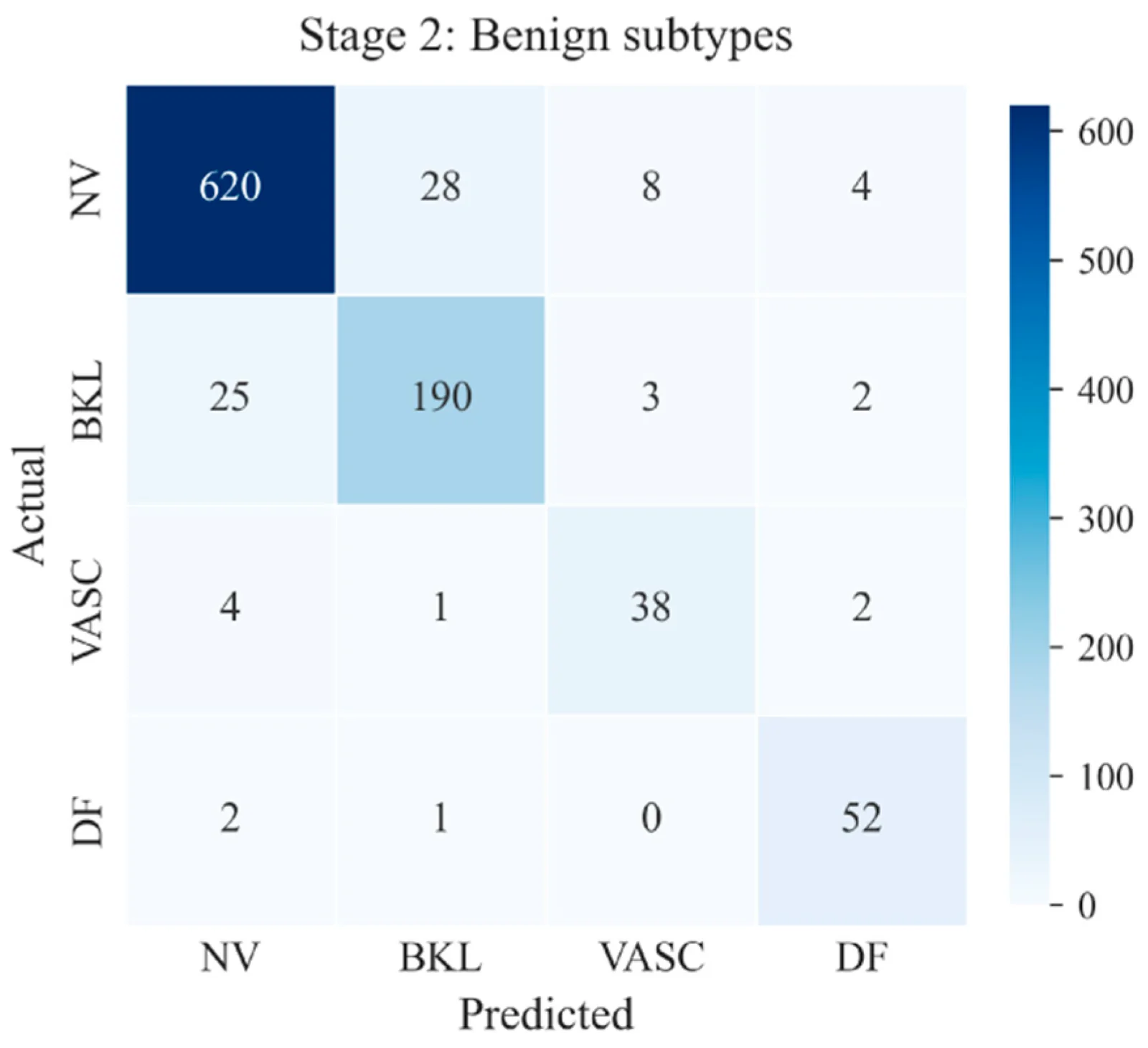
Representative test-set confusion matrix of the Stage 2 benign subtype classifier.

The benign branch achieved 91.84 ± 0.41% overall accuracy, 86.60 ± 1.37% precision, 89.48 ± 0.89% recall, and 87.97 ± 1.13% F1-score (Table 7). Although the accuracies for BKL, VASC, and DF may be influenced by their relatively small sample sizes, NV exhibits separability with a high volume of correctly classified cases (Figure 10). Both Stage 2 malignant and benign models effectively distinguish among their subtypes, including certain challenging categories such as AKIEC, SCC, VASC, and DF that have much smaller sample sizes and higher intra-class variability.

Taken together, the results demonstrate that MorphoNet provides a hierarchical classification strategy for both benign–malignant screening and fine-grained subtype recognition. The combination of EfficientNet-B4 visual features, ABCD-guided attention, and CORAL regularization supports both predictive performance and model interpretability across the integrated dermoscopic dataset.

## 5. Conclusions

This study introduced MorphoNet, a hierarchical deep learning model for automated dermoscopic skin lesion classification. The proposed approach combines EfficientNet-B4 with quantitative ABCD morphological descriptors through an attention-guided feature fusion strategy and incorporates CORAL-based domain alignment to improve robustness across multiple datasets. By separating benign–malignant screening from subtype classification, the model follows a diagnostic workflow inspired by routine clinical practice while providing interpretable information through learned attention weights.

Evaluation on a combined dataset of HAM10000, ISIC 2019, and DERM12345 showed that MorphoNet achieved high accuracy for both binary screening and lesion subtype classification. The learned attention patterns were generally consistent with established dermatologic assessment criteria, indicating that the model captures clinically relevant morphological characteristics while maintaining strong classification performance across heterogeneous data sources. These results support the value of integrating domain knowledge with deep learning for more interpretable skin lesion analysis.

Several limitations should be acknowledged. MorphoNet was evaluated only on pooled public datasets and has not yet been validated using an entirely independent clinical cohort. Residual class imbalance, particularly for AKIEC, SCC, DF, and VASC, may affect subtype-classification performance, while the learned attention weights should be interpreted as explanatory signals for the model rather than direct clinical evidence. Future work will focus on independent multicenter validation and improved learning strategies for underrepresented lesion categories.

## Figures and Tables

**Table 1:** Class-wise distribution of the images used in this study.

	MEL	BCC	AKIEC	SCC	NV	BKL	DF	VASC	Total
HAM10000	1113	514	130	197	6703	1099	115	142	10,013
ISIC 2019	2275	1766	729	431	0	893	124	111	6329
DERM12345	0	0	58	266	0	0	179	0	503
Total	3388	2280	917	894	6703	1992	418	253	16,845

**Table 2:** ABCD features used in Stage 1 and Stage 2 models.

Category	Features	Feature Index	Description
A—Asymmetry	Basic	asym_score, asym_h, asym_v	overall, horizontal, and vertical measurements
	Advanced	asym_ratio, asym_entropy	horizontal/vertical asymmetry ratio and entropy measurement
B—Border	Basic	perimeter_area_ratio, boundary_complexity, circularity	Border irregularity and complexity values
	Advanced	compactness, fractal_dim	shape regularity, compactness, and fractal dimension
C—Color	RGB variance	color_variance_r/g/b,	RGB channel color variations
	HSV variance	color_variance_h/s/v	HSV channel color variations
	Color entropy	hist_entropy_r/g/b	RGB histogram entropy for color diversity
	Color diversity	color_diversity	overall color diversity combining all variance
D—Diameter	Size measures	diameter_feature, diameter_ratio, normalized_diameter	Pixel-space and image-normalized lesion-size measurements

**Table 3:** Component-wise ablation results of MorphoNet.

Classification Task	Model Configuration	Accuracy	Precision	Recall	F1 Score
Stage 1	Visual only	91.84 ± 0.72	91.26 ± 0.81	91.14 ± 0.79	91.18 ± 0.80
Stage 1	+ABCD	93.08 ± 0.61	92.71 ± 0.69	92.62 ± 0.67	92.65 ± 0.68
Stage 1	+ABCD + CORAL	94.01 ± 0.50	93.73 ± 0.56	93.68 ± 0.55	93.70 ± 0.55
Stage 2: malignant	Visual only	85.73 ± 0.88	79.31 ± 1.44	80.02 ± 1.38	79.52 ± 1.41
Stage 2: malignant	+ABCD	87.58 ± 0.69	81.66 ± 1.19	82.18 ± 1.16	81.84 ± 1.18
Stage 2: malignant	+ABCD + CORAL	89.05 ± 0.51	83.75 ± 1.02	84.34 ± 0.99	84.00 ± 1.00
Stage 2: benign	Visual only	88.93 ± 0.79	81.84 ± 1.88	85.44 ± 1.34	83.26 ± 1.60
Stage 2: benign	+ABCD	90.47 ± 0.58	84.36 ± 1.60	87.41 ± 1.10	85.53 ± 1.33
Stage 2: benign	+ABCD + CORAL	91.84 ± 0.41	86.60 ± 1.37	89.48 ± 0.89	87.97 ± 1.13

**Table 4:** Comparison of MorphoNet with representative image-based baseline models.

Classification Task	Model	Accuracy	Precision	Recall	F1 Score
Stage 1	ResNet-50	90.31 ± 0.88	89.62 ± 0.94	89.47 ± 0.91	89.51 ± 0.92
Stage 1	DenseNet-121	90.96 ± 0.81	90.31 ± 0.86	90.22 ± 0.84	90.24 ± 0.85
Stage 1	EfficientNet-B4	91.84 ± 0.72	91.26 ± 0.81	91.14 ± 0.79	91.18 ± 0.80
Stage 1	Full MorphoNet	94.01 ± 0.50	93.73 ± 0.56	93.68 ± 0.55	93.70 ± 0.55
Stage 2: malignant	ResNet-50	83.71 ± 1.07	76.32 ± 1.66	77.10 ± 1.58	76.57 ± 1.61
Stage 2: malignant	DenseNet-121	84.62 ± 0.96	77.76 ± 1.54	78.54 ± 1.47	78.02 ± 1.50
Stage 2: malignant	EfficientNet-B4	85.73 ± 0.88	79.31 ± 1.44	80.02 ± 1.38	79.52 ± 1.41
Stage 2: malignant	Full MorphoNet	89.05 ± 0.51	83.75 ± 1.02	84.34 ± 0.99	84.00 ± 1.00
Stage 2: benign	ResNet-50	86.94 ± 0.98	78.86 ± 2.02	82.5 ± 1.55	80.27 ± 1.76
Stage 2: benign	DenseNet-121	87.86 ± 0.89	80.35 ± 1.94	83.92 ± 1.46	81.71 ± 1.68
Stage 2: benign	EfficientNet-B4	88.93 ± 0.79	81.84 ± 1.88	85.44 ± 1.34	83.26 ± 1.60
Stage 2: benign	Full MorphoNet	91.84 ± 0.41	86.60 ± 1.37	89.48 ± 0.89	87.97 ± 1.13

**Table 5:** Final test metrics of the Stage 1 binary model across three random seeds.

Models	Types	Accuracy	Precision	Recall	F1 Score
Stage 1	Malignant	94.01 ± 0.50	92.38 ± 0.65	92.22 ± 0.74	92.30 ± 0.70
	Benign	94.01 ± 0.50	95.08 ± 0.48	95.14 ± 0.35	95.11 ± 0.41
	Overall	94.01 ± 0.50	93.73 ± 0.56	93.68 ± 0.55	93.70 ± 0.55

**Table 6:** Final test metrics of the Stage 2 malignant model across three random seeds.

Models	Types	Accuracy	Precision	Recall	F1 Score
Stage 2 Malignant	MEL	93.93 ± 0.28	94.39 ± 0.47	94.15 ± 0.12	94.27 ± 0.25
	BCC	95.27 ± 0.22	90.23 ± 0.68	90.47 ± 0.25	90.35 ± 0.43
	AKIEC	94.82 ± 0.32	70.69 ± 1.25	74.53 ± 1.85	72.57 ± 1.56
	SCC	94.16 ± 0.43	82.69 ± 1.83	78.20 ± 1.80	80.37 ± 1.81
	Overall	89.05 ± 0.51	83.75 ± 1.02	84.34 ± 0.99	84.00 ± 1.00

**Table 7:** Final test metrics of the Stage 2 benign model across three random seeds.

Models	Types	Accuracy	Precision	Recall	F1 Score
Stage 2 Benign	NV	92.77 ± 0.59	95.26 ± 0.43	93.94 ± 0.46	94.59 ± 0.44
	BKL	93.91 ± 0.41	86.48 ± 0.90	86.36 ± 0.91	86.42 ± 0.91
	VASC	98.16 ± 0.21	77.96 ± 2.71	83.70 ± 1.28	80.72 ± 1.96
	DF	98.85 ± 0.15	86.69 ± 1.45	93.94 ± 1.05	90.16 ± 1.22
	Overall	91.84 ± 0.41	86.60 ± 1.37	89.48 ± 0.89	87.97 ± 1.13

## Data Availability

Data are contained within the article.
